# Is Dispositional Self-Compassion Associated With Psychophysiological Flexibility Beyond Mindfulness? An Exploratory Pilot Study

**DOI:** 10.3389/fpsyg.2020.00614

**Published:** 2020-04-09

**Authors:** Julie Lillebostad Svendsen, Elisabeth Schanche, Berge Osnes, Jon Vøllestad, Endre Visted, Ingrid Dundas, Helge Nordby, Per-Einar Binder, Lin Sørensen

**Affiliations:** ^1^Division of Psychiatry, Haukeland University Hospital, Bergen, Norway; ^2^Department of Biological and Medical Psychology, Faculty of Psychology, University of Bergen, Bergen, Norway; ^3^Department of Clinical Psychology, Faculty of Psychology, University of Bergen, Bergen, Norway; ^4^Bjørgvin District Psychiatric Centre, Haukeland University Hospital, Bergen, Norway; ^5^Solli District Psychiatric Centre, Nesttun, Norway

**Keywords:** self-compassion, mindfulness, heart rate variability, young adults, anxiety, rumination

## Abstract

**Background:**

Dispositional mindfulness and self-compassion are shown to associate with less self-reported emotional distress. However, previous studies have indicated that dispositional self-compassion may be an even more important buffer against such distress than dispositional mindfulness. To our knowledge, no study has yet disentangled the relationship between dispositional self-compassion and mindfulness and level of psychophysiological flexibility as measured with vagally mediated heart rate variability (vmHRV). The aim was thus to provide a first exploratory effort to expand previous research relying on self-report measures by including a psychophysiological measure indicative of emotional stress reactivity.

**Methods:**

Fifty-three university students filled out the “Five Facet Mindfulness Questionnaire” (FFMQ) and the “Self-Compassion Scale” (SCS), and their heart rate was measured during a 5 min resting electrocardiogram. Linear hierarchical regression analyses were conducted to examine the common and unique variance explained by the total scores of the FFMQ and the SCS on level of resting vmHRV.

**Results:**

Higher SCS total scores associated significantly with higher levels of vmHRV also when controlling for the FFMQ total scores. The SCS uniquely explained 7% of the vmHRV. The FFMQ total scores did not associate with level of vmHRV.

**Conclusion:**

These results offer preliminary support that dispositional self-compassion associates with better psychophysiological regulation of emotional arousal above and beyond mindfulness.

## Introduction

How we relate to our experiences and ourselves when dealing with difficult emotions is important for our psychological health (Gross and Munoz, 1995). Dispositional mindfulness and self-compassion are two related and inherent human capacities (Kabat-Zinn, 2003). Mindfulness is a multi-faceted construct that can be defined as “paying attention in a particular way; on purpose, in the present moment, and non-judgmentally” (Kabat-Zinn, 2003). Self-compassion emphasizes being mindful to one’s own suffering together with the active wish to alleviate it and relate to oneself with a kind and caring attitude (Neff, 2003b). It is described as a positive mental capacity especially important for the health promoting outcomes associated with mindfulness-based interventions (Van Dam et al., 2011) in mediating decreased levels of symptoms of depression (Kuyken et al., 2010) and stress responses (Shapiro et al., 2005). Mindfulness and self-compassion are partially overlapping in their emphasis on non-judgment, acceptance and tolerance. At the same time, self-compassion focuses more exclusively on suffering, and more explicitly emphasizes kindness for the self (Neff and Dahm, 2015). This could capture a distinct dimension of human experience that can be meaningfully separated from mindfulness, and that may also be particularly important for dealing with challenging emotions.

To disentangle dispositional self-compassion from mindfulness, self-report measures are typically used (Kuyken et al., 2010; Van Dam et al., 2011; Woodruff et al., 2014). A widely employed mindfulness scale is the Five Facet Mindfulness Questionnaire (FFMQ; Baer et al., 2006), measuring five distinct mindful aspects: observing, describing, acting with awareness, non-judgment, and non-reactivity to inner experiences. This measure is reckoned to include both attentional and attitudinal components of being mindful, in contrast to another widely use scale called the Mindful Attention and Awareness Scale (MAAS; Brown and Ryan, 2003), which exclusively focuses on present moment attention. The most commonly used self-report measure of self-compassion is the Self-Compassion Scale (SCS; Neff, 2003a), distinguishing between self-kindness versus self-judgment, common humanity versus isolation, and mindfulness versus overidentification. Thus, as operationally defined by Neff (2003a), mindfulness is an embedded part of self-compassion, however, this form of mindfulness is more narrowly focused on suffering, whereas mindfulness as measured by the FFMQ taps a broader range of experiences – both positive, neutral and negative ones.

A handful of studies have compared the predictive values of dispositional mindfulness and self-compassion on self-reported outcome measures. Van Dam et al. (2011) focused on the common and unique variance of dispositional mindfulness as measured with the MAAS and dispositional self-compassion with the SCS on self-reported symptoms of anxiety, depression, worry, and life quality. They found that both constructs were significant predictors, however, self-compassion uniquely explained up to ten times more variance of positive outcomes on these measures than mindfulness. Woodruff et al. (2014) sought to replicate these findings using the multi-faceted mindfulness-measure FFMQ. Similar to Van Dam et al. (2011) they found that both mindfulness and self-compassion significantly explained lower levels of anxiety, depression, negative affect, and unhappiness, and that self-compassion uniquely explained a higher degree of variance in these outcome variables than mindfulness. The central role of dispositional self-compassion as a buffer against emotional problems has further been shown in relation to level of shame (Woods and Proeve, 2014) and post-traumatic symptoms (Dahm et al., 2015), in self-compassion being a stronger predictor of lower levels of these symptoms than mindfulness. Interestingly, a recent study also showed self-compassion to predict emotional flexibility (Beshai et al., 2017). It showed that only self-compassion predicted better abilities in mood drop and spontaneous mood-recovery when including both mindfulness (measured with the MAAS) and self-compassion (measured with the SCS) as predictors in the same regression model.

Dispositional mindfulness (e.g., Delgado-Pastor et al., 2013; Krygier et al., 2013; Nijjar et al., 2014; Watford and Stafford, 2015; May et al., 2016; Shearer et al., 2016) and dispositional self-compassion (Rockliff et al., 2008; Kok et al., 2013; Arch et al., 2014; Matos et al., 2017; Petrocchi et al., 2017) are further also linked to psychophysiological flexibility as measured with higher vagally mediated heart rate variability (vmHRV). However, as far as we know, no study has yet attempted to disentangle the effect of dispositional self-compassion from dispositional mindfulness on vmHRV. Higher vmHRV is used as a psychophysiological index of the ability to regulate emotional arousal in flexible and adaptive ways according to situational contexts (Thayer and Lane, 2000, 2009; Appelhans and Luecken, 2006; Holzman and Bridgett, 2017). Resting vmHRV is suggested to reflect about 50% of trait-like aspects of the typical interplay between the sympathetic and parasympathetic branches of the autonomic nervous system (Bertsch et al., 2012). The sympathetic nervous system (SNS) increases heart rate, whereas the parasympathetic nervous system (PNS) slows down heart rate via the vagus nerve. The PNS has a shorter latency of response, thus allowing for higher variability between heartbeats. Higher resting vmHRV variability reflects as such a predominant PNS stimulation on the beat-to-beat heart rate. Lack of flexibility in the up- and down regulation of mood and emotional responses, i.e., emotional arousal, can be reflected in a lower vmHRV (Appelhans and Luecken, 2006). Previous research has shown that higher levels of symptoms of anxiety (Chalmers et al., 2014), depression (Kemp et al., 2010), and worry (Chalmers et al., 2016) are associated with lower levels of vmHRV. Our research group found that dispositional self-compassion as measured with the SCS associated with higher vmHRV, measured both during rest and during 24-h, and that this positive effect of self-compassion on vmHRV remained when adjusting for the effects of symptoms of anxiety and negative rumination (Svendsen et al., 2016). Similarly, it has been shown that individuals high in dispositional self-compassion have higher vmHRV in response to an acute stressor than individuals low in self-compassion (Luo et al., 2018). It has also been shown that self-compassion training increases vmHRV (Rockliff et al., 2008; Kok et al., 2013; Arch et al., 2014; Matos et al., 2017; Petrocchi et al., 2017). To our knowledge, only a few studies have investigated the relationship between dispositional mindfulness and vmHRV. One of these (Prazak et al., 2012) found that university students reporting higher levels of dispositional mindfulness on the Kentucky Inventory of Mindfulness Skills had higher vmHRV than those reporting to be less mindful. Two studies have found mixed results of the relationship between mindfulness as measured with the MAAS and vmHRV. One (Mankus et al., 2013) only found a positive association between high dispositional mindfulness on the MAAS and high vmHRV among individuals reporting to be high on symptoms of general anxiety, and not among those reporting to be low on general anxiety symptoms. The other (Brzozowski et al., 2017) found no association between reports on the MAAS and vmHRV. Fogarty et al. (2015) found only an association between the FFMQ total scores and level of vmHRV after an emotional stress induction task in men but not women. Likewise, Kadziolka et al. (2016) found the FFMQ-subscales of “Observe” and “Acting with Awareness” to relate to a higher vmHRV after a stress induction task. Interestingly, in contrast to measuring dispositional mindfulness, a more consistent relationship is found between mindfulness training and increased levels of vmHRV (e.g., Takahashi et al., 2005; Ditto et al., 2006; Tang et al., 2009; Joo et al., 2010; Delgado-Pastor et al., 2013; Krygier et al., 2013; Nijjar et al., 2014; Azam et al., 2015; Watford and Stafford, 2015; May et al., 2016; Shearer et al., 2016), although one study did not find such an effect of mindfulness training on level of vmHRV (Nyklicek et al., 2013).

In the present study the aim was to explore if dispositional self-compassion related with vmHRV above and beyond mindfulness. Several authors have emphasized the importance of measuring peoples’ dispositional levels, since pre-existing dispositional mindfulness may influence what is considered an effect of mindfulness training (such as when using vmHRV as an effect measure; Tang et al., 2016; Wheeler et al., 2016; Zhuang et al., 2017). The use of self-report instrument of dispositional self-compassion with SCS and mindfulness with FFMQ allowed for comparison of the effects found on vmHRV with previous findings using self-reported symptoms as outcome variables (e.g., Van Dam et al., 2011; Woodruff et al., 2014). However, the experimental design of relying solely on self-reports in a small sample of healthy adults and including only resting vmHRV as a trait-measure of psychophysiological flexibility may limit the generalizability of the findings. The objective was therefore to conduct an explorative pilot study in which the results can be important for generating hypotheses for future studies. We primarily explored the common and unique explained variance of dispositional self-reported mindfulness and self-compassion on level of resting vmHRV. A secondary aim was to replicate previous findings that self-compassion explained variance beyond mindfulness on self-reported distress symptoms of anxiety and negative rumination.

## Materials and Methods

### Participants and Procedure

Participants were recruited through ads and posters to the student population of the University of Bergen, Norway (see also Svendsen et al., 2016; Visted et al., 2017; Sorensen et al., 2018, 2019). Initially we included 56 participants, but three participants were excluded due to poor data quality on the vmHRV-measures. Thus, the sample consisted of 53 students (36 female, mean age = 23.6 years, SD = 2.52; mean body mass index (BMI) = 21.80, SD = 2.33).

Written informed consent was obtained from all participants, in accordance with the Helsinki declaration. The protocol was approved by the Regional Committees for Medical and Health Research Ethics – South East Norway, Gullhaugveien 1-3, 0318 Oslo (study number 2014/148). Exclusion criteria were usage of sedative or psychoactive medication, heart conditions, and previous experience with mindfulness, i.e., attendance at mindfulness courses, retreats, or other kinds of formalized mindfulness instruction.

When participants arrived on the testing day, they received a detailed explanation of the tests involved, but no information regarding hypotheses of the study. They were then seated in a room in which they were asked to fill out a package of questionnaires, including the SCS, the FFMQ, the Rumination Reflection Questionnaire (RRQ), the State-Trait Anxiety Questionnaire, as well as information about age, gender, and BMI. One at a time, they were invited to move to an experimental room, in order to record their heart rate with an electrocardiogram (ECG). Participants were asked not to exercise, smoke or drink caffeine six hours prior to the experiment.

Participants were offered a brief and intensive mindfulness course free of charge, and were not offered any additional compensation for participation in the study.

### Measures

#### Heart Rate Variability

The ECG was recorded with a standard lead-II setup and digitized at 1000 Hz. Data from all participants were collected at approximately the same time in the afternoon in order to control for circadian effects. The signal was obtained through an A/D converter (Biopac, MP36, BIOPAC System, Inc., Santa Barbara, CA, United States) recorded with Biopac 4.0 BSL (BIOPAC Systems, Inc., Santa Barbara, CA, United States). VmHRV was assessed during a 5-min period in which the participants were asked to find a position that felt comfortable and relax as much as possible.

Heart-rate data were manually checked for artifacts (movement, electrode noise, and extraordinary peaks) offline, and then subjected to a vmHRV analysis with Kubios version 2.0 (Tarvainen et al., 2014). In the current study, we used the high frequency (HF) power measured in milliseconds as a measure of vmHRV. HF is considered to be a valid measure of vmHRV (Task force, 1996; Thayer and Sternberg, 2006; Li et al., 2009; Williams et al., 2015), and is the most commonly used in previous research investigating the relationship between self-compassion, mindfulness and vmHRV (Takahashi et al., 2005; Tang et al., 2009; Delgado-Pastor et al., 2013; Kok et al., 2013; Krygier et al., 2013; Nyklicek et al., 2013; Nijjar et al., 2014; Azam et al., 2015; Watford and Stafford, 2015; May et al., 2016). The HF measure correlated highly (*r* = 0.94, *p* > 0.001) with the time domain Root-mean square of successive R-R intervals (RMSSD), also a measure of vagus-mediated autonomic influence on the heart (Task force, 1996; Shaffer and Ginsberg, 2017). Additional correlations between the RMSSD and the total scores of the FFMQ and the SCS are included in Supplementary Material.

All vmHRV data were subjected to a HRV analysis with Kubios version 2.0 (Tarvainen et al., 2014), and HF and RMSSD was calculated. Trend components were removed with the smoothness priors detrending method (λ = 500).

#### Self-Compassion Scale

The SCS (Neff, 2003a) consists of 26 items reflecting three positive and three negative subscales. The positive subscales are: self-kindness (e.g., “I try to be loving toward myself when I’m feeling emotional pain.”), common humanity (e.g., “When things are going badly for me, I see the difficulties as part of life that everyone goes through”), and mindfulness (e.g., “When something upsets me I try to keep my emotions in balance”). The negative subscales are: self-judgment (e.g., “When times are really difficult, I tend to be tough on myself”), isolation (e.g., “When I think about my inadequacies, it tends to make me feel more separate and cut off from the rest of the world”), and over-identification (e.g., “When I’m feeling down I tend to obsess and fixate on everything that’s wrong”). Items are rated on a five-point Likert-type scale where 1 equals “almost never” and 5 equals “almost always.” Low scores on the negative subscales and high scores on the positive subscales reflect an overall high level of self-compassion.

The SCS has been shown to have good reliability and cross-cultural validity (Neff et al., 2008). We employed a Norwegian validated translation of the SCS (Dundas et al., 2016; see Supplementary Material to article for validation of the scale in Norwegian).

#### Five Facet Mindfulness Questionnaire

The FFMQ (Baer et al., 2006) is a 39 items self-report measure of mindfulness. Items are rated on a five-point Likert -type scale from 1 (“never or very rarely true”) to 5 (“very often or always true”), measuring five factors: observing (e.g., “I notice the smells and aromas of things”), describing (e.g., “I am good at finding words to describe my feelings”), acting with awareness (e.g., “I find myself doing things without paying attention”; reverse scored item), non-judging of inner experience (e.g., “I think some of my emotions are bad or inappropriate and I should not feel them”; reverse scored item), and non-reactivity to inner experience (e.g., “I perceive my feelings and emotions without having to react to them”).

The FFMQ has shown high construct validity (Baer et al., 2006, 2008). The five subscales are internally consistent, with alpha coefficients ranging from 0.76 to 0.91 (Baer et al., 2006). The test-retest reliability was demonstrated to be good to excellent in a Dutch sample (Veehof et al., 2011). In the present study we used a Norwegian validated translation of the FFMQ (Dundas et al., 2013).

#### Trait Subscale of the State-Trait Anxiety Inventory

In order to measure trait anxiety, we used the Trait subscale of the State-Trait Anxiety Inventory (STAI; Spielberger et al., 1983), consisting of 20 items. Examples of items are “I am a steady person” (reversed) and “I have disturbing thoughts.” Scores are rated on a four point Likert scale ranging from 1 (”almost never”) to 4 (”almost always”). The Trait scale of the STAI has shown excellent internal consistency (average α < 0.89) and test-retest reliability (average *r* = 0.88; Barnes et al., 2002). In the present study we used a Norwegian translation of the STAI (Spielberger et al., 1983).

#### Rumination Subscale of the Rumination Reflection Questionnaire

Rumination was measured using the 12-item Rumination subscale of the RRQ (Trapnell and Campbell, 1999). Example items are “Long after an argument or disagreement is over with, my thoughts keep going back to what happened” and “I often find myself re-evaluating something I have done.” Answers are rated on a five-point Likert-type scale ranging from 1, indicating “strongly disagree”, to 5, equaling “strongly agree”. We employed a Norwegian validated translation of the RRQ-Rum scale (Verplanken et al., 2007).

### Statistical Analysis

All vmHRV measures were log transformed as to approximate a normal distribution. The data were statistically analyzed using SPSS version 24.0. Pearson bivariate correlational analyses were conducted to test the relationship between vmHRV, the SCS scores, and the FFMQ scores, respectively. In order to ensure that the bivariate relationships observed were not confounded by shared variance between the total scores from the FFMQ and the SCS, two linear hierarchical regression analyses were run to test the common and unique explained variance of the total scores of the FFMQ and the SCS, respectively, on the level of vmHRV. To determine the unique explained variance of the FFMQ and the SCS, respectively, the FFMQ was in the first regression model entered in the first step and the SCS in the second step, and thereafter, in the second regression model, the SCS was entered in the first step and the FFMQ in the second step (i.e., with *F* analysis of stepwise change in explained variance). In these hierarchical linear regression models, effects were adjusted for level of BMI (Koenig et al., 2014) and gender (Koenig and Thayer, 2016) on vmHRV. Further, the common and unique explained variance of the total scores of the FFMQ and the SCS, respectively, were also tested with linear hierarchical regression models with self-reported levels of anxiety and negative rumination, respectively, as outcome variables. These results are briefly presented in the section “Results,” and tables can be found in Supplementary Material.

No outliers were detected (±3 standard deviations from the sample mean). Missing item scores for six participants were replaced by sample mean for each item. The skewness and kurtosis levels for FFMQ, SCS, and vmHRV were within border limits (*z* > 1.96 or *z* < −1.96; Field, 2013).

## Results

In the current study, the level of self-compassion (SCS) ranged from 1.31 to 4.27, with a mean level of 2.78 (SD = 0.83). The level of mindfulness (FFMQ) ranged from 2.03 to 4.74, with a mean of 3.04 (SD = 0.64). The SCS total score and the FFMQ total score demonstrated excellent internal consistencies with Cronbach’s alpha coefficients of 0.96 and 0.95, respectively. The level of trait anxiety (trait subscale of the STAI) ranged from 20 to 69 with a mean of 43.75 (SD = 12.58), and the level of rumination (rumination subscale of the RRQ) ranged from 14.0 to 57.0, with a mean level of 43.71 (SD = 10.58). The trait subscale of the STAI and the rumination subscale of the RRQ evidenced excellent internal consistencies with Cronbach’s alphas of 0.95 and 0.93, respectively.

There was a significant correlation between the total scores of the SCS and the FFMQ (see Table 1). Only the SCS total score, and not the FFMQ total score, correlated significantly with level of vmHRV (see also Supplementary Table 1). Higher SCS total scores correlated with higher levels of vmHRV. There was no significant correlation between vmHRV and the demographic variables of BMI, gender, and age.

**TABLE 1:** Bivariate correlations between vmHRV, SCS, and FFMQ total scores, and covariates.

1	2	3	4	5	6
1. vmHRV (HF)	0.29*	0.17	0.07	–0.20	0.05
2. SCS total		0.68**	0.16	0.22	0.27*
3. FFMQ total			0.19	0.21	0.15
4. Age				–0.03	0.07
5. Gender					–0.01
6. BMI					

*N* = 53. *p < 0.05; **p < 0.01. vmHRV (HF), high frequency vagally mediated heart rate variability; SCS, Self-Compassion Scale; total score, FFMQ, Five Facet Mindfulness Questionnaire, total score.

In hierarchical linear regression models, only the SCS total scores, and not the FFMQ total scores, significantly explained variance of the level of vmHRV (see Table 2). The FFMQ total scores did not significantly explain vmHRV independently of whether the scores were included in the first or second step of the model. The SCS total scores explained 7.3% of the level of vmHRV when controlling for the level of the FFMQ total scores in the first step of the model, and explained 11.7% when not controlling for the level of the FFMQ total scores (the SCS total scores were included in the first step). The covariate of gender predicted significantly level of vmHRV in the second step of the regression models when both the total scores of the FFMQ and the SCS were included as predictors. Level of BMI did not affect level of vmHRV as a covariate in the regression models.

**TABLE 2:** Hierarchical regression analysis of the relation between FFMQ/SCS and vmHRV.

Total sample	Model	Step	Predictor	*R*^2^	Δ*R*^2^	*df*	Δ*F*	β step 3
vmHRV (HF):	1	0	Gender	0.04	0.04	2/50	1.09	−0.28*
(*N* = 53)			BMI					–0.06
		1	FFMQ	0.09	0.04	1/49	2.37	–0.03
		2	SCS	0.16	0.07	1/48	4.17*	0.38*
	2	1	SCS	0.16	0.12	1/49	6.81*	0.38*
		2	FFMQ	0.16	0.00	1/48	0.02	–0.03

*N* = 53. *p < 0.05. FFMQ, Five Facet Mindfulness Questionnaire; SCS, Self-Compassion Scale; vmHRV (HF), high frequency vagally mediated heart rate variability.

Linear hierarchical regression analyses showed that both the total scores from the FFMQ and the SCS significantly explained lower levels of self-reported trait anxiety and negative rumination when entered in the first step of the model, respectively (see Supplementary Table 2). The SCS total scores explained higher amounts of variance than the FFMQ total scores, whether entered in the first or second step. The FFMQ total scores only showed a trend for significantly explaining higher levels of trait anxiety when controlling for the effect of SCS total scores (by entering the FFMQ total scores in the second step).

## Discussion

The present pilot study provided preliminary results indicating that dispositional self-compassion is a stronger predictor of vmHRV than mindfulness. Self-compassion as measured with the total scores of the SCS associated uniquely with higher vmHRV when controlling for the effect of mindfulness as measured by the total scores of the FFMQ. This suggests that being more self-compassionate associates with a more flexible modulation of physiological responses compared to being low on self-compassion. We also found as previous research relying on self-report measures of emotional distress (e.g., Van Dam et al., 2011; Woodruff et al., 2014) that the total scores of the SCS explained more variance than the total scores of the FFMQ on self-report measures of anxiety and negative rumination. The main contribution of the present study was thus to expand previous research with self-reports by including a psychophysiological measure indicative of trait-like emotional stress reactivity.

Our preliminary findings suggest that dispositional self-compassion is an important psychological buffer against emotional stress. It seems to provide emotional safety when suffering and may also be related to increased self-efficacy that one will be able to provide oneself with the emotional support and warmth needed in times of difficulty (Finlay-Jones, 2017). Although mindfulness and self-compassion are deeply interconnected and reciprocally facilitate each other (Baer, 2010; Neff and Dahm, 2015), it has been hypothesized that self-compassion is distinguishable from mindfulness in several ways that may be particularly important for stress regulation. Self-compassion seems to be applicable predominantly in times of suffering, whereas mindfulness taps the ability to be present with a much broader specter of experiences (i.e., not only difficult experiences, but also positive and neutral ones; Germer, 2009; Neff and Dahm, 2015). Moreover, self-compassion seems to focus more on common humanity; the recognition that suffering is a normal part of life and that everyone fails sometimes (Neff and Dahm, 2015). Finally, self-compassion more explicitly emphasizes self-soothing (Neff and Dahm, 2015). This ability to soothe and comfort oneself is indeed theoretically proposed to be connected to vagal activity (Porges, 2007), leading to higher physiological flexibility across situations. Therefore, the current study has followed the recommendation of compassion research to include vmHRV as a primary outcome measure. Higher vmHRV may as such represent the physiological signature of a trait-like compassionate responding (Kirby et al., 2017).

Interestingly, we found that dispositional mindfulness as measured by the FFMQ was not significantly associated with level of vmHRV. It appears that there is stronger evidence for trained mindfulness to predict higher vmHRV (e.g., Ditto et al., 2006; Delgado-Pastor et al., 2013; Nijjar et al., 2014; Shearer et al., 2016) compared to dispositional mindfulness (e.g., Prazak et al., 2012; Mankus et al., 2013). Some of the studies that have found a significant association between dispositional mindfulness and level of vmHRV have measured heart rate under stress contingencies (Fogarty et al., 2015; Kadziolka et al., 2016) or included participants with high levels of anxiety symptoms (Mankus et al., 2013). In the present study we focused on resting vmHRV since this is the condition that have been highlighted to be a trait-marker of flexibility in psychophysiological regulation (Thayer and Lane, 2009; Thayer et al., 2009). However, it is shown that resting vmHRV explains about 50% of trait-like aspects of personality (Bertsch et al., 2012). This means that resting vmHRV will also reflect state aspects, which for instance can be influenced by the context of the test condition and each participant’s reaction to this context. Future studies are as such encouraged to study the common and unique associations between mindfulness and self-compassion and level of vmHRV also under stress conditions or after stress modulation.

It is possible that effects of mindfulness are best captured by measuring effects of training rather than using self-reports (e.g., Tang et al., 2015). However, mindfulness training is shown to increase self-compassion (Shapiro et al., 2005; Kuyken et al., 2010), therefore effects of mindfulness training can encompass also increased levels of self-compassion. In process-oriented research there is a wish to disentangle effects of self-compassion from mindfulness *per se* (Kuyken et al., 2010). Then the applied questionnaires of the FFMQ and the SCS are the most commonly used in the literature examining these concepts, despite them being under scrutiny on psychometrical grounds (Park et al., 2013; Williams et al., 2014; Strauss et al., 2016 see also Grossman and Van Dam, 2011). Thus, important to note, despite self-reports being a common method used in research investigating effects of self-compassion and mindfulness on mental health, it can be biased information (Grossman, 2011; Kazdin, 2014). For instance, it might be easier for participants to self-report on positive attitudes toward oneself than it is to self-report frequency of past mindful states (Van Dam et al., 2011). In fact, it has been raised concerns that mindfulness taps into higher-order cognitive processes that are challenging for individuals to be aware of and to report on in questionnaires (Van Dam et al., 2011; see also Nisbett and Wilson, 1977). Therefore, future studies can address this limitation by designing and/or using experimental conditions independently assessing self-compassion and mindfulness. For instance, a recent study of self-compassion and vmHRV (Kirschner et al., 2019) showed that an experimental condition of self-compassion was superior in predicting higher vmHRV compared to conditions not representing self-compassion.

The experimental design of the present study has several limitations that future studies are encouraged to address. As already mentioned, research has shown that the state component in resting vmHRV is greater than previously thought, encouraging the inclusion of experimentally conditioned states recordings such as a stress-induction (see Bertsch et al., 2012). The correlational design does not allow for causal interpretations, which could have been addressed by including repeated measures of vmHRV and/or self-compassion and ECG recordings during exercises of mindfulness and self-compassion. Also, we have followed the recommendation of about 5 min ECG recordings (Task force, 1996), which may be less reliable than applying longer recordings (Bourdillon et al., 2017). The use of self-reports could have been complemented by also including experimental conditions of self-compassion and mindfulness. Finally, the present pilot study included a small sample consisting of young, healthy and well-functioning students, which may not be representative of other populations. The statistical power may as such have been restricted to detect effects of for instance dispositional mindfulness on vmHRV. This can be observed in that the explained variance (*R*^2)^ indicated a small effect size of the model when including the two related concepts of dispositional self-compassion and mindfulness as predictors of vmHRV.

## Conclusion

In summary, the present pilot study suggests that dispositional self-compassion associates with better psychophysiological regulation of emotional arousal compared to mindfulness. Taking into account the explorative nature of this study, the results point to self-compassion having the potential to provide a buffer against psychophysiological stress reactivity and supports targeting self-compassion in clinical work. The findings correspond to recent developments in the clinical mindfulness field, toward increasing the explicit focus of self-compassion in mindfulness-based interventions (Feldman and Kuyken, 2011; Segal et al., 2013). Future studies are encouraged to continue shedding light on the unique and common benefits of being self-compassionate and mindful.

## Data Availability Statement

The datasets generated for this study are available on request to the corresponding author.

## Ethics Statement

The participants gave informed consent in accordance with the Helsinki declaration, and the protocol was approved by the Regional Ethics Committee (South-East, Study number 2014/148).

## Author Contributions

LS and ES: project leaders. JS, ES, BO, JV, EV, HN, P-EB, and LS: conception and design. JS, ES, BO, JV, EV, and LS: collection of the data. JS and LS: analysis and interpretation, and writing of the manuscript. ES, BO, JV, EV, ID, HN, and P-EB: critical review of the manuscript. JS, ES, BO, JV, EV, ID, HN, P-EB, and LS: final approval for publication.

## Conflict of Interest

The authors declare that the research was conducted in the absence of any commercial or financial relationships that could be construed as a potential conflict of interest.
