# Supplementary material for: Is Dispositional Self-Compassion Associated With Psychophysiological Flexibility Beyond Mindfulness? An Exploratory Pilot Study
Source: Front Psychol. 2020 Apr 9;11:614. doi: 10.3389/fpsyg.2020.00614 (PMC7160328; doi:10.3389/fpsyg.2020.00614)
Supplement: Supplementary file 1 [file Table_1.docx]

Supplementary Material

**An exploratory pilot study: Is dispositional self-compassion associated with psychophysiological flexibility beyond mindfulness?**

Svendsen, J.L.^*^, Schanche, E., Osnes, B., Vøllestad, J., Visted, E., Dundas, I., Nordby, H., Binder, P.-E., & Sørensen, L.

*** Correspondence:** Corresponding Author: [**julie.svendsen@uib.no**](mailto:julie.svendsen@uib.no)

# Supplementary Tables

**Supplemental Table 1: Bivariate correlations between RMSSD, SCS and FFMQ**

Note: N=53. *p<.05; **p<.01. SCS= Self-Compassion Scale; FFMQ= Five Facet Mindfulness Questionnaire; vmHRV (RMSSD)= Root mean square of successive differences, vagally mediated heart rate variability
