# Supplementary material for: Is Dispositional Self-Compassion Associated With Psychophysiological Flexibility Beyond Mindfulness? An Exploratory Pilot Study
Source: Front Psychol. 2020 Apr 9;11:614. doi: 10.3389/fpsyg.2020.00614 (PMC7160328; doi:10.3389/fpsyg.2020.00614)
Supplement: Supplementary file 2 [file Table_2.docx]

Supplementary Material

**An exploratory pilot study: Is dispositional self-compassion associated with psychophysiological flexibility beyond mindfulness?**

Svendsen, J.L.^*^, Schanche, E., Osnes, B., Vøllestad, J., Visted, E., Dundas, I., Nordby, H., Binder, P.-E., & Sørensen, L.

*** Correspondence:** Corresponding Author: [**julie.svendsen@uib.no**](mailto:julie.svendsen@uib.no)

# Supplementary Tables

**Supplemental Table 2: Hierarchical regression analysis of the relation between FFMQ/SCS and Trait Anxiety/ Rumination**

|  |  |  |  |  |  |  |  |  |
| --- | --- | --- | --- | --- | --- | --- | --- | --- |
| **Total sample** | **Model** | **Step** | **Predictor** | ***R2*** | ***ΔR2*** | ***df*** | ***ΔF*** | ***β* step 2** |
| **STAI-Trait** | 1 | 1 | FFMQ | .43 | .43 | 1/51 | 39.04** | -.21a |
| (n=53) |  | 2 | SCS | .67 | .23 | 1/50 | 34.52** | -.66** |
|  | 2 | 1 | SCS | .64 | .64 | 1/51 | 91.43** | -.66** |
|  |  | 2 | FFMQ | .67 | .02 | 1/50 | 3.43a | -.21a |
| **RRQ-Rum** | 1 | 1 | FFMQ | .41 | .41 | 1/51 | 35.29** | -.26* |
| (n=53) |  | 2 | SCS | .57 | .16 | 1/50 | 19.15** | -.55** |
|  | 2 | 1 | SCS | .54 | .54 | 1/51 | 59.03** | -.55** |
|  |  | 2 | FFMQ | .57 | .04 | 1/50 | 4.22* | -.26* |
|  |  |  |  |  |  |  |  |  |

Note: N=53. * p<.05; **p<.01; a: p=.07. FFMQ= Five Facet Mindfulness Questionnaire; SCS= Self-Compassion Scale; STAI-Trait= Trait subscale of the State-trait anxiety inventory; RRQ-rum= Rumination subscale of the Rumination-Reflection Questionnaire
